# An updated framework for characterizing patients with pediatric feeding disorder

**DOI:** 10.3389/frcha.2025.1653288

**Published:** 2025-09-15

**Authors:** Valerie M. Volkert, Collen T. Lukens, Alan H. Silverman, Laura Johnson, William G. Sharp

**Affiliations:** ^1^ Department of Pediatrics, Emory University, Atlanta, GA, United States; ^2^ Department of Child and Adolescent Psychiatry, The Children’s Hospital of Philadelphia, Philadelphia, PA, United States; ^3^ Medical College of Wisconsin, Milwaukee, WI, United States

**Keywords:** feeding, case report form, pediatric feeding disorder, patient characterization, feasibility study

## Abstract

Expert consensus previously established a framework for characterizing patients with pediatric feeding disorder (PFD) through a multidisciplinary case report form (CRF) methodology. Field testing of the PFD CRF and creation of a shared patient database represented next steps in the development of this clinical tool. The current study assessed the acceptability and feasibility of the PFD CRF through data collection across three feeding programs involved in the initial development of the CRF. A total of 80 patients completed multidisciplinary team evaluations and contributed data to the current evaluation of clinical implementation. Data analysis and feedback from end users (i.e., multidisciplinary care teams) subsequently guided CRF revisions. Results suggests the PFD CRF represents a feasible and practical method of common data collection across institutions, while also providing important insights into future research and dissemination efforts.

## Introduction

Historically, lack of clarity surrounding the diagnosis of a feeding disorder across health care professionals impacted timely identification, communication, and optimal treatment pathways. Goday et al. proposed a conceptual definition of pediatric feeding disorder (PFD) using the World Health Organization International Classification of Functioning, Disability, and Health (ICF) framework that considers the interaction of factors across medical, nutritional, feeding skill, and psychosocial domains each of which can cause or contribute to a disruption in oral intake (1). The International Classification of Diseases 10th Revision adopted the uniform definition of PFD in 2019 (2).

In 2022, Sharp et al. (3) developed a standardized data collection system to characterize patients with PFD as a necessary first step in promoting methodologically sound research (3). This involved convening an expert panel from multidisciplinary programs within the United States to develop common data elements for a PFD case report form (CRF). The subsequent PFD CRF is intended to promote a standard method for patient characterization, enhance methodological rigor, and provide a useful clinical tool for clinicians and researchers working with this pediatric population. The next step proposed by Sharp et al. with the PFD CRF focused on assessing the feasibility of data collection through field testing (3, 4).

The current study completed field testing of the PFD CRF across three multidisciplinary locations in the United States [Children's Healthcare of Atlanta [CHOA], Children's Hospital of Philadelphia [CHOP], Medical College of Wisconsin [MCW]] (3). The primary purpose was to prospectively assess acceptability and feasibility related to implementation of the PFD CRF by multidisciplinary provider teams and evaluate completeness of data collection. Based implementation data, a secondary purpose was to further revise and finalize the PFD CRF for wider distribution.

## Materials and methods

The current study involved three major activities. First, the study team reviewed and made modifications to the CRF in preparation for implementation. This process involved the multidisciplinary teams at the three participating centers evaluating the usability of the items as part of routine clinical care by CRF domain (medical, nutrition, feeding skill, psychosocial). In preparation for testing, changes consisted of minor modifications in wording, organization/flow of items, and deletion and/or addition of items. Prior to field testing, domain leads trained the clinical teams across sites.

Second, each program sought to prospectively collect data using the PFD CRF for 30 participants (total of 90) between January 17, 2023 to June 14, 2023. Eligibility criteria included patients aged 1–21 years who completed a full multidisciplinary team evaluation (medical, psychology, feeding skill, and nutrition) and the availability of a study team member (i.e., principal investigator; research assistant) to consent the family at the time of the appointment. Patients with a single discipline evaluation (e.g., only nutrition) or who were non-English speaking (at MCW only) were excluded. Study team members completed data collection for the CRF during the clinical appointment and finalized data entry through a review of the electronic health record for that encounter. Third, analysis of data collected during field testing and feedback from the discipline specific subgroups guided an update to the CRF. The Institutional Review Board of each participating institution approved the study protocol. Respondents gave written consent for review and signature before data collection.

### Measurement

The PFD CRF used during field testing mirrored the structure outlined by Sharp et al. (3), with items divided into the four PFD domains: Medical, Nutrition, Feeding Skill, and Psychosocial. We also developed a demographic section to support this project. The CRFs consisted of 65 questions from the four domains and of those, twenty-seven were considered stem questions. A respondent's answer to a stem question triggered display logic for subsequent questions. For example, a positive response for disorders that affect oral, nasal, or pharyngeal function resulted in a series of questions to ask if the disorder was tethered tissue released (yes/no), macroglossia (yes/no), labial or palatal clefts (yes/no), etc.

### Data analysis

To evaluate acceptability and feasibility of the CRFs, each location assessed willingness of patients to participate (target: no more than 12.5% refusal rate and drop-out rate <15% once enrolled), capability of the provider team to collect 95% of the data (target: missingness 5% or <), as well as an analysis of data quality and completeness of items.

Analysis of missing data focused on the survey questions evaluating the four domains of PFD. For each respondent, the proportion of missing data was calculated as follows:

$$\mathrm{Proportion}\;\mathrm{Missing}=1\text{–}\left(\frac{\#\mathrm{of}\;\mathrm{Questions}\;\mathrm{Answered}\;}{\#\mathrm{of}\;\mathrm{Questions}\;\mathrm{Seen}\;}\right)=1-\left(\frac{\;\mathrm{Total}\;\#\;\mathrm{of}\;\mathrm{Questions}\text{–}\mathrm{NA}\;\text{–}\mathrm{HQ}-\mathrm{NR}}{\mathrm{Total}\;\#\;\mathrm{of}\;\mathrm{Questions}\text{–}\mathrm{NA}\;\text{–}\;\mathrm{HQ}\;}\right)$$

The proportion missing was summarized for the overall sample (*N* = 80 respondents). Then, to evaluate variability, proportion missing was compared across domains (medical, nutrition, feeding skill, and psychosocial) and sites (CHOA, CHOP, MCW).

## Results

### Data collection and patient population

Parents of 94 children were invited to participate in the study across the three sites. Of those invited to participate, 7 did not meet eligibility criteria and 7 declined to participate (Supplementary Figure S1). This resulted in an 8% refusal rate from apparently eligible participants, 80 enrolled participants, and a drop-out rate of 0% once enrolled. The respective research teams were able to collect 98% of the data. Mean missingness of the data (Table 1) was low overall, by domain, and across the three multidisciplinary sites (4% or less). Two of the three sites met enrollment targets; MCW ceased data collection after 20 participants due logistical issues.

**Table 1:** Proportion missing data: overall and by CRF domain, & site.

	Min–Max	Mean	Median (IQR)
Overall	0–0.13	0.02	0.02 (0.01, 0.04)
By domain			
Medical	0–0.69	0.03	0.02 (0.02, 0.02)
Nutrition	0–0.24	0.04	0.02 (0, 0.07)
Feeding Skill	0–0.14	0.03	0.03 (0, 0.03)
Psychosocial	0–0.12	0.006	0 (0, 0)
By site			
CHOA	0.02–0.13	0.04	0.04 (0.03, 0.04)
CHOP	0–0.08	0.01	0.008 (0.005, 0.01)
Wisconsin	0–0.06	0.02	0.03 (0.01, 0.03)

The patient's mother (85% of respondents) was most often the respondent. Most patients were under 6 years of age (73%) with the most common age range between 3 and 5 (46%). The sample involved 57 males (71%) and 23 females (29%) and caregivers reported White (49%) or African American (22%) as the most common racial background. The only statistically significant difference across the three locations was related to racial and ethnic background. The patients at MCW were less diverse and predominantly White while the patients at CHOP were more likely to be Hispanic/Latino.

#### Updated PFD CRF

Nine item level changes were made to the CRF. In the medical domain, this included removing *Intubation* from the Pregnancy and Birth section, adding *inflammatory bowel disease* to Gastrointestinal disorders/Food reactions, adding the response option major interruption to typical feeding progress due to medication under the stem Iatrogenic, and creating a separate section for Allergy/Food Intolerance conditions. In the nutrition domain, removing Evidence of nutrition inadequacy as support by diet record because this information is duplicative with raw data on food groups and a new stem Vitamin was added, with *multivitamin with iron*, *multivitamin without iron*, *iron*, *calcium*, *vitamin D*, and *other* representing subitem options. Two subitems were added to the feeding skill domain—i.e., *breastfeeding/chestfeeding* and *infant bottle*—under drinking format. Revisions in the psychosocial domain involved adding a subitem to now distinguish between *externalizing* and *internalizing* problem behaviors outside of meals under the stem *Behavioral/Developmental Complexity*. Other minor changes focused on enhancing the operational definitions in the corresponding protocol to improve the reliability of data collection. The updated PFD CRF is provided in Supplementary Figures S2–S5.

## Discussion

A CRF framework supports methodological rigor in clinical research by promoting complete and accurate data collection (5). Previous accounts of patients with PFD involve descriptive, retrospective single-site studies lacking uniform data collection (3). Expert consensus guided development of the PFD CRF and field testing represented an important next toward broader dissemination. The current study piloted data collection across three sites to determine ease of implementation, completeness of data collection, and generalizability of use to multidisciplinary providers at each site. Our findings show that use of the CRF was feasible for both clinicians and families, with participation rate and completeness of data were high, 92% and 96%, respectively. Furthermore, efforts to build an inter-intuitional data base appears feasible as data were shared across centers.

Recent estimates suggest PFD is exceedingly common, occurring at a prevalence rate between 1 in 23 to 1 in 37 children under the age of 5 (6). Thus, healthcare providers are frequently called upon to help families find appropriate assessment and treatment resources. Children with PFD benefit from multidisciplinary care, particularly in complex cases (1, 3). Yet many providers lack the knowledge and resources to manage these problems. Moving forward, dissemination efforts should include training for providers on the use of the PFD CRF in line with the need for more general education efforts on the complex etiology of PFD. Ideally, this will also facilitate enhanced awareness of this relatively new diagnosis and promote referrals to specialists who are best suited to manage this multifaceted condition.

Researchers included only 80 English-speaking individuals receiving a multidisciplinary assessment in the United States which may limit the generalizability of the findings and broader applicability to patients in other countries. Data collection was also restricted to three locations which may not fully capture variability in measurement across other healthcare systems. The PFD CRF requires multidisciplinary teams and specialized training, which may be challenging for smaller or less-resourced programs. Future research should compare the acceptability and feasibility of the CRF to other existing tools for patient characterization (e.g., Pediatric Eating Assessment Tool) and expand data collection to include other multidisciplinary centers to both promote a more comprehensive representation of patients and increase inter-site collaborations through a shared data base (7). Determining etiological underpinnings is another important area for further inquiry and future investigators should incorporate the PFD CRF with other assessment methods to understand this complex and heterogeneous condition.

Clinically, the PFD CRF will support gathering reliable data for analysis and comparison and may be useful to evaluate changes in patient presentations over the course of clinical care. Better patient characterization could ultimately help clinicians identify which treatments are optimal for which patients and improve clinical outcomes. Finally, while the purpose of the current study was not intended to establish psychometric properties of the CRF (reliability and validation), further work on this process may yield support for its use as a research tool with established psychometric properties.

## Data Availability

The raw data supporting the conclusions of this article will be made available by the authors, without undue reservation.
