# Supplementary material for: An updated framework for characterizing patients with pediatric feeding disorder
Source: Front Child Adolesc Psychiatry. 2025 Sep 15;4:1653288. doi: 10.3389/frcha.2025.1653288 (PMC12477044; doi:10.3389/frcha.2025.1653288)

Supplementary Figure 1. Flow Diagram of Participants who Enrolled in Multisite Feasibility Trial

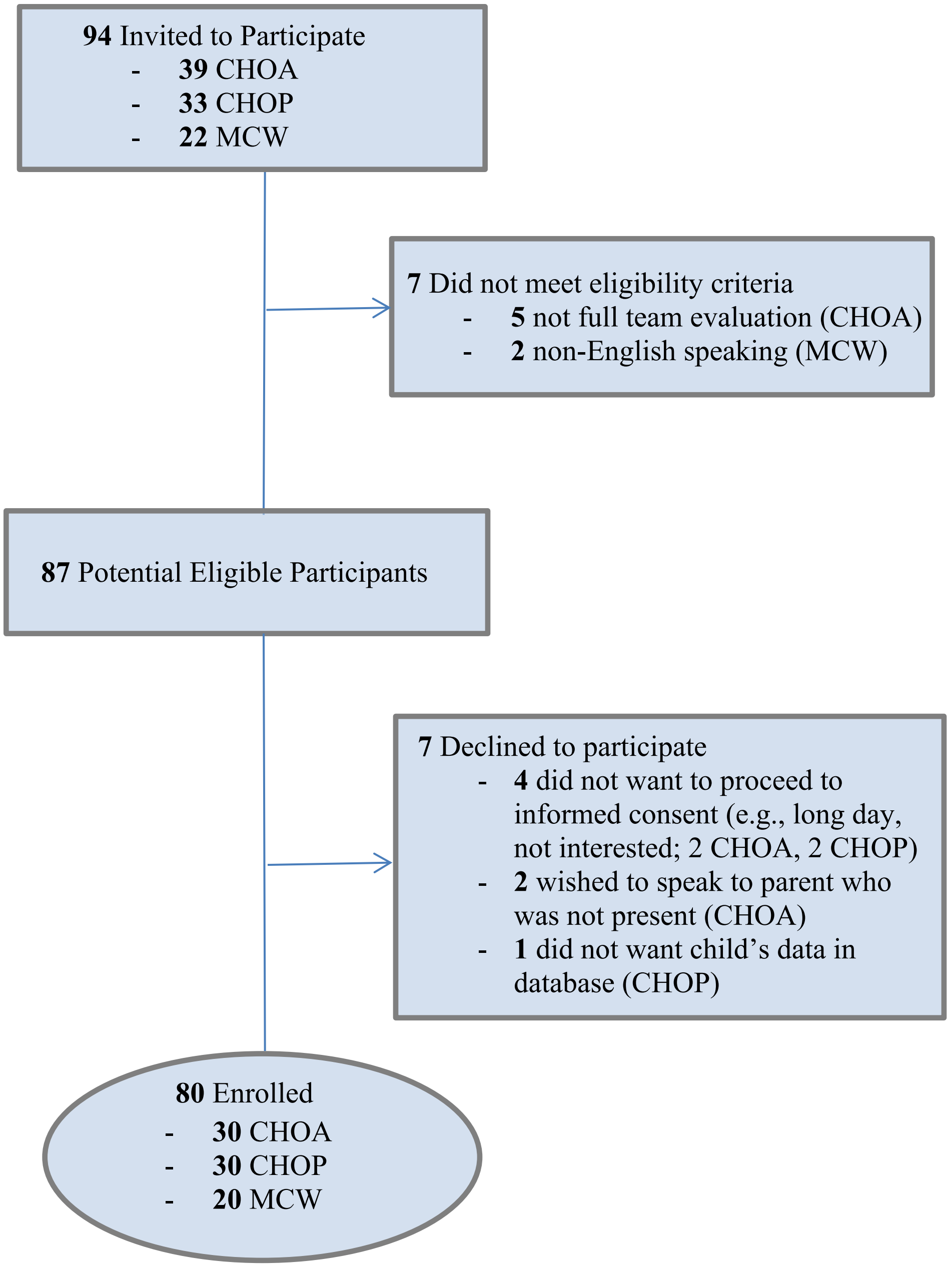

Supplement: Supplementary file 1 [file Image1.pdf]
