# Supplementary material for: An updated framework for characterizing patients with pediatric feeding disorder
Source: Front Child Adolesc Psychiatry. 2025 Sep 15;4:1653288. doi: 10.3389/frcha.2025.1653288 (PMC12477044; doi:10.3389/frcha.2025.1653288)
Supplement: Supplementary file 2 [file Image2.pdf]

Supplementary Figure 2. Medical Case Report Form

SECTION 1: MEDICAL DOMAIN	
**1.1 Pregnancy and Birth**	
**1.1.a Chronological age**	☐☐ months/years | ☐ NR (9)
**1.1.b Gestational age**	☐☐ weeks | ☐ NR (9)
**1.1.c NICU**	☐ Yes (1) ☐ No (0) ☐ NR (9)
**1.2 Medical Diagnoses**	
**1.2.a Disorders that affect oral, nasal, or pharyngeal function**	☐ Yes (1) ☐ No (0) – If no, skip to 1.2.b ☐ NR (9)
Tethered tissue released	☐ Yes (1) ☐ No (0) ☐ NR (9)
Macroglossia	☐ Active (2) ☐ Resolved (1) ☐ Never (0) ☐ NR (9)
Extensive dental disease	☐ Active (2) ☐ Resolved (1) ☐ Never (0) ☐ NR (9)
Labial or palatal clefts	☐ Active (2) ☐ Resolved (1) ☐ Never (0) ☐ NR (9)
Velopharyngeal insufficiency	☐ Active (2) ☐ Resolved (1) ☐ Never (0) ☐ NR (9)
Choanal atresia/pirform aperture stenosis	☐ Active (2) ☐ Resolved (1) ☐ Never (0) ☐ NR (9)
Tonsillar hypertrophy	☐ Active (2) ☐ Resolved (1) ☐ Never (0) ☐ NR (9)
Adenoid hypertrophy	☐ Active (2) ☐ Resolved (1) ☐ Never (0) ☐ NR (9)
**1.2.b Airway Disorders**	☐ Yes (1) ☐ No (0) – If no, skip to 1.2.c ☐ NR (9)
Laryngeal cleft	☐ Active (2) ☐ Resolved (1) ☐ Never (0) ☐ NR (9)
Vocal fold paralysis or injury	☐ Active (2) ☐ Resolved (1) ☐ Never (0) ☐ NR (9)
Airway malacia (laryngo-, tracheo-, or bronchomalacia)	☐ Active (2) ☐ Resolved (1) ☐ Never (0) ☐ NR (9)
Airway stenosis	☐ Active (2) ☐ Resolved (1) ☐ Never (0) ☐ NR (9)
Tracheoesophageal fistula	☐ Active (2) ☐ Resolved (1) ☐ Never (0) ☐ NR (9)
**1.2.c Pulmonary disorders**	☐ Yes (1) ☐ No (0) – If no, skip to 1.2.d ☐ NR (9)
Bronchopulmonary dysplasia	☐ Active (2) ☐ Resolved (1) ☐ Never (0) ☐ NR (9)
Chronic respiratory failure requiring mechanical ventilation	☐ Active (2) ☐ Resolved (1) ☐ Never (0) ☐ NR (9)
Aspiration or recurrent aspiration pneumonias	☐ Active (2) ☐ Resolved (1) ☐ Never (0) ☐ NR (9)
**1.2.d Gastrointestinal disorders/Food reactions**	☐ Yes (1) ☐ No (0) – If no, skip to 1.2.e ☐ NR (9)
Eosinophilic esophagitis	☐ Active (2) ☐ Resolved (1) ☐ Never (0) ☐ NR (9)
Esophageal dysmotility	☐ Active (2) ☐ Resolved (1) ☐ Never (0) ☐ NR (9)
Esophageal stricture	☐ Active (2) ☐ Resolved (1) ☐ Never (0) ☐ NR (9)
H. Pylori	☐ Active (2) ☐ Resolved (1) ☐ Never (0) ☐ NR (9)
Volume intolerance of any cause	☐ Active (2) ☐ Resolved (1) ☐ Never (0) ☐ NR (9)
Constipation	☐ Active (2) ☐ Resolved (1) ☐ Never (0) ☐ NR (9)
Intestinal failure	☐ Active (2) ☐ Resolved (1) ☐ Never (0) ☐ NR (9)
Inflammatory Bowel Disease	☐ Active (2) ☐ Resolved (1) ☐ Never (0) ☐ NR (9)
Gastroesophageal reflux disease	☐ Active (2) ☐ Resolved (1) ☐ Never (0) ☐ NR (9)
**1.2.e Congenital cardiac disease and other heart disease**	☐ Yes (1) ☐ No (0) – If no, skip to 1.2.f ☐ NR (9)
Any form of congenital heart disease (esp. hypoplastic left heart syndrome) and other	☐ Active (2) ☐ Resolved (1) ☐ Never (0) ☐ NR (9)

conditions that result in staged single ventricle repair	
Associated pulmonary hypertension	☐ Active (2) ☐ Resolved (1) ☐ Never (0) ☐ NR (9)
Heart failure	☐ Active (2) ☐ Resolved (1) ☐ Never (0) ☐ NR (9)
Surgical intervention for cardiac disease	☐ Yes (1) ☐ No (0) ☐ NR (9)
**1.2.f Neurological, developmental, and psychiatric disorders**	☐ Yes (1) ☐ No (0) – If no, skip to 1.2.g ☐ NR (9)
Autism spectrum disorder	☐ Yes (1) ☐ No (0) ☐ NR (9)
Attention deficit/hyperactivity disorder	☐ Yes (1) ☐ No (0) ☐ NR (9)
Anxiety disorder	☐ Yes (1) ☐ No (0) ☐ NR (9)
Cerebral palsy	☐ Yes (1) ☐ No (0) ☐ NR (9)
Abnormal tone	☐ Yes (1) ☐ No (0) ☐ NR (9)
Developmental delay	☐ Yes (1) ☐ No (0) ☐ NR (9)
Muscular dystrophies	☐ Yes (1) ☐ No (0) ☐ NR (9)
Chromosomal abnormality	☐ Yes (1) ☐ No (0) ☐ NR (9)
Specify condition(s):	______________________________________
**1.2.g Iatrogenic**	☐ Yes (1) ☐ No (0) – If no, skip to 1.2.h ☐ NR (9)
Major interruption to typical feeding progress due to procedure	☐ Active (2) ☐ Resolved (1) ☐ Never (0) ☐ NR (9)
Major interruption to typical feeding progress due to medication	☐ Active (2) ☐ Resolved (1) ☐ Never (0) ☐ NR (9)
**1.2.h Allergy/Food Intolerance**	☐ Yes (1) ☐ No (0) – If no, skip to 1.3 ☐ NR (9)
Asthma	☐ Active (2) ☐ Resolved (1) ☐ Never (0) ☐ NR (9)
Eczema	☐ Active (2) ☐ Resolved (1) ☐ Never (0) ☐ NR (9)
FPIES (Food protein-induced enterocolitis syndrome)	☐ Active (2) ☐ Resolved (1) ☐ Never (0) ☐ NR (9)
Food Allergy (IgE mediated)	☐ Active (2) ☐ Resolved (1) ☐ Never (0) ☐ NR (9)
Lactose intolerance	☐ Active (2) ☐ Resolved (1) ☐ Never (0) ☐ NR (9)
**1.3 Procedures & Tests**	☐ Yes (1) ☐ No (0) ☐ NR (9)
Swallow Study (MBS/OPMS/VFSS)	☐ Yes (1) ☐ No (0) ☐ Unknown (3) ☐ NR (9)
Results:	
Fiberoptic evaluation of swallowing (FEES)	☐ Yes (1) ☐ No (0) ☐ Unknown (3) ☐ NR (9)
Results:	
Direct Laryngoscopy	☐ Yes (1) ☐ No (0) ☐ Unknown (3) ☐ NR (9)
Results:	
Upper GI Endoscopy (Esophagogastroduodenoscopy/EGD)	☐ Yes (1) ☐ No (0) ☐ Unknown (3) ☐ NR (9)
Results:	
24-hour Impedance probe study	☐ Yes (1) ☐ No (0) ☐ Unknown (3) ☐ NR (9)
Results:	
Gastrointestinal motility testing	☐ Yes (1) ☐ No (0) ☐ Unknown (3) ☐ NR (9)
Results:	
Fundoplication	☐ Yes (1) ☐ No (0) ☐ Unknown (3) ☐ NR (9)
Results:	
Tracheostomy	☐ Yes (1) ☐ No (0) ☐ Unknown (3) ☐ NR (9)
Results:	
**1.4 Medications to Promote Hunger**	☐ Yes (1) ☐ No (0) ☐ NR (9)

SECTION 1: MEDICAL DOMAIN PROTOCOL	
Item	Detail
1.1	Pregnancy and birth history
1.1a	Chronological age listed in months if under 3 years of age and in years if over 3 (do not include additional months)
1.1.b	Gestational age in weeks
1.1.c	Indicate yes or no for NICU stay
1.2	Medical Diagnoses
1.2.a	Indicate yes or no for history of or presence of disorders affecting oral, nasal, or pharyngeal function including conditions that cause difficulty controlling a bolus in the mouth (e.g., ankyloglossia), cause obstruction of the nasal cavity/nasopharynx (e.g., enlarged adenoids or nasal cavity narrowing), or cause impairment of pharyngeal contraction, often due to deconditioning or weakness.<br><br>For specific conditions:<br>o Macroglossia - if Trisomy 21 or Beckwith-Wiedemann endorse “Active”; If not endorse “Never”<br>o Tonsillar hypertrophy – endorse “Active” from exam and/or from noting consultation from specialty providers for this condition; if history of tonsillectomy endorse “Resolved”<br>o Adenoid hypertrophy - endorse “Active” if had imaging or ENT had concerns; if history of adenoidectomy endorse “Resolved”
1.2.b	Indicate yes or no for history of or presence of disorders impacting the airway, which may challenge the suck-swallow-breath coordination in young children during feeding. It may also involve conditions that result in a narrowing of the airway (i.e., stenosis) or weak cartilage (malacia), which may necessitate a tracheostomy to bypass stenosis and malacia.
1.2.c	Indicate yes or no for history of or presence of pulmonary disorders
1.2.d	Indicate yes or no for history of or presence of gastrointestinal disorders. For specific conditions:<br>o Esophageal dysmotility - endorse only if had manometry that was abnormal<br>o Volume intolerance of any cause – endorse if cannot get to goal volume without vomiting, or history of need for post pyloric feed, or vomiting when exceed target oral volume<br>o Gastroesophageal reflux<br>• Do not endorse if only infant spit ups, infant use of Zantac<br>• Endorse if frequently documented in chart or actively noted by other medical providers
1.2.e	Indicate yes or no for history of or presence of congenital heart disease or cardiac surgery.
1.2.f	Indicate yes or no for history of or presence of neurological, developmental, and psychiatric conditions. Endorse only if diagnosed.
1.2.g	Indicate yes or no for history of or presence of illness caused by medical examination or treatment. As applied to a feeding disorder, iatrogenic disease may be related to a medical event that interrupts the development of feeding skill or limits/restricts oral intake (e.g., tongue-lip adhesion).<br><br>- Procedure based: endorse if a child had been eating typically but could not eat for a long period of time due to procedure (e.g., surgical intervention)

	- Medication based: Endorse if medication induced anorexia is observed (e.g., chemotherapy, psychostimulants)
1.2.h	Allergy/Food Intolerance<br>Includes asthma, eczema, FPIES, and food allergy/intolerance
1.3	Indicate yes or no for history of assessment procedures include procedures examining the pharynx and esophagus for swallow safety (i.e., swallow study, FEES), observing the airway and upper GI (e.g., laryngoscopy, EGD), assisting in diagnosis of acid reflux (24 hour Impedance probe study), and detecting concerns regarding GI transit (Gastrointestinal motility testing).
1.4	Indicate yes or no for current use of medications to promote hunger such as Cyproheptadine (Periactin) at the time of intake
