# Supplementary material for: An updated framework for characterizing patients with pediatric feeding disorder
Source: Front Child Adolesc Psychiatry. 2025 Sep 15;4:1653288. doi: 10.3389/frcha.2025.1653288 (PMC12477044; doi:10.3389/frcha.2025.1653288)
Supplement: Supplementary file 3 [file Image3.pdf]

SECTION 2: NUTRITION DOMAIN		
**2.1 Calorie Breakdown**		
**2.1.a Total Calories**	☐☐☐☐ Kcals ☐ NR (9)	
**2.1.b Calories from tube**	☐☐☐☐ Kcals ☐ NR (9)	
**2.1.c Calories from formula taken orally**	☐☐☐☐ Kcals ☐ NR (9)	
**2.1.d Calories from food consumed orally**	☐☐☐☐ Kcals ☐ NR (9)	
**2.1.e Calories from fluid that is not formula**	☐☐☐☐ Kcals ☐ NR (9)	
**2.1.f Estimated caloric needs**	☐☐☐☐ Kcals ☐ NR (9)	
**2.2 Primary Source of Nutrition**	i. Tube Feeding	☐ Yes (1) ☐ No (0) ☐ NR (9)
	ii. Drinking Formula	☐ Yes (1) ☐ No (0) ☐ NR (9)
	iii. Oral Consumption	☐ Yes (1) ☐ No (0) ☐ NR (9)
**2.3 Nutritional Supplementation**		
**2.3.a Current Supplementation**	☐ Yes (1) ☐ No (0) – If no, skip to 2.4 ☐ NR (9)	
**2.3.b Percent Supplementation**	☐☐ ☐ percent of needs from supplementation ☐ NR (9) ☐ NA (8)	
**2.3.c Reason for supplementation**	☐ Promote growth (1) ☐ To address nutritional needs (2) ☐ To support hydration(3) ☐ NA (8) ☐ NR (9)	
**2.3.d Source of supplementation**	☐ Tube (1) ☐ Oral Formula (2) ☐ IV Fluids (3) ☐ NA (8) ☐ NR (9)	
**2.4 Dietary Diversity**		
**2.4.a.i Vegetables**	☐ Yes (1) ☐ No (0) – If no, skip to 2.4.b ☐ NR (9)	
**2.4.a.ii Estimated # of vegetables**	☐ 0 (0) | ☐ 1-2 (1) | ☐ 3-4 (2)| ☐ 5 + (3) | ☐ NR (9)	
**2.4.b.i Fruits**	☐ Yes (1) ☐ No (0) – If no, skip to 2.4.c ☐ NR (9)	
**2.4.b.ii Estimated # of fruits**	☐ 0 (0) | ☐ 1-2 (1) | ☐ 3-4 (2)| ☐ 5 + (3) | ☐ NR (9)	
**2.4.c.i Dairy/Dairy Alternative**	☐ Yes (1) ☐ No (0) – If no, skip to 2.4.d ☐ NR (9)	
**2.4.c.ii Estimated # of dairy/dairy alternative**	☐ 0 (0) | ☐ 1-2 (1) | ☐ 3-4 (2)| ☐ 5 + (3) | ☐ NR (9)	
**2.4.d.i Proteins**	☐ Yes (1) ☐ No (0) – If no, skip to 2.4.e ☐ NR (9)	
**2.4.d.ii Estimated # of protein**	☐ 0 (0) | ☐ 1-2 (1) | ☐ 3-4 (2)| ☐ 5 + (3) | ☐ NR (9)	
**2.4.e.i Grains**	☐ Yes (1) ☐ No (0) – If no, skip to 2.4.f ☐ NR (9)	
**2.4.e.ii Estimated # of grains**	☐ 0 (0) | ☐ 1-2 (1) | ☐ 3-4 (2)| ☐ 5 + (3) | ☐ NR (9)	
**2.4.f.i Sweets/snack**	☐ Yes (1) ☐ No (0) – If no, skip to 2.5 ☐ NR (9)	
**2.4.f.ii Estimated # of sweets/snacks**	☐ 0 (0) | ☐ 1-2 (1) | ☐ 3-4 (2)| ☐ 5 + (3) | ☐ NR (9)	
**2.5 Evidence of nutritional deficiency as supported by laboratory analysis**	☐ Yes (1) ☐ No (0) ☐ NR (9) ☐ NA (8)	
**Nutrient**	**Deficiency**	
**2.5.a Energy**	☐ Yes (1) ☐ No (0) ☐ NR (9) ☐ NA (8)	
**2.5.b Carbohydrate**	☐ Yes (1) ☐ No (0) ☐ NR (9) ☐ NA (8)	
**2.5.c Protein**	☐ Yes (1) ☐ No (0) ☐ NR (9) ☐ NA (8)	
**2.5.d Fat**	☐ Yes (1) ☐ No (0) ☐ NR (9) ☐ NA (8)	
**2.5.e Vitamin A**	☐ Yes (1) ☐ No (0) ☐ NR (9) ☐ NA (8)	
**2.5.f Vitamin B12**	☐ Yes (1) ☐ No (0) ☐ NR (9) ☐ NA (8)	
**2.5.g Vitamin C**	☐ Yes (1) ☐ No (0) ☐ NR (9) ☐ NA (8)	

**2.5.h Vitamin D**	☐ Yes (1) ☐ No (0) ☐ NR (9) ☐ NA (8)
**2.5.i Vitamin E**	☐ Yes (1) ☐ No (0) ☐ NR (9) ☐ NA (8)
**2.5.j Folic Acid**	☐ Yes (1) ☐ No (0) ☐ NR (9) ☐ NA (8)
**2.5.k Calcium**	☐ Yes (1) ☐ No (0) ☐ NR (9) ☐ NA (8)
**2.5.l Iron**	☐ Yes (1) ☐ No (0) ☐ NR (9) ☐ NA (8)
**2.5.m Zinc**	☐ Yes (1) ☐ No (0) ☐ NR (9) ☐ NA (8)
**2.5.n Total nutrients <80 DRI**	☐ ☐ | ☐ NR (9) ☐ NA (8)
**2.6 Evidence of nutritional deficiency as supported by analysis of 24-hour recall**	☐ Yes (1) ☐ No (0) ☐ NR (9) ☐ NA (8)
**Nutrient**	**Deficiency**
**2.6.a Energy**	☐ Yes (1) ☐ No (0) ☐ NR (9) ☐ NA (8)
**2.6.b Carbohydrate**	☐ Yes (1) ☐ No (0) ☐ NR (9) ☐ NA (8)
**2.6.c Protein**	☐ Yes (1) ☐ No (0) ☐ NR (9) ☐ NA (8)
**2.6.d Fat**	☐ Yes (1) ☐ No (0) ☐ NR (9) ☐ NA (8)
**2.6.e Vitamin A**	☐ Yes (1) ☐ No (0) ☐ NR (9) ☐ NA (8)
**2.6.f Vitamin B12**	☐ Yes (1) ☐ No (0) ☐ NR (9) ☐ NA (8)
**2.6.g Vitamin C**	☐ Yes (1) ☐ No (0) ☐ NR (9) ☐ NA (8)
**2.6.h Vitamin D**	☐ Yes (1) ☐ No (0) ☐ NR (9) ☐ NA (8)
**2.6.i Vitamin E**	☐ Yes (1) ☐ No (0) ☐ NR (9) ☐ NA (8)
**2.6.j Folic Acid**	☐ Yes (1) ☐ No (0) ☐ NR (9) ☐ NA (8)
**2.6.k Calcium**	☐ Yes (1) ☐ No (0) ☐ NR (9) ☐ NA (8)
**2.6.l Iron**	☐ Yes (1) ☐ No (0) ☐ NR (9) ☐ NA (8)
**2.6.m Zinc**	☐ Yes (1) ☐ No (0) ☐ NR (9) ☐ NA (8)
**2.6.n Total nutrients <80 DRI**	☐ ☐ | ☐ NR (9) ☐ NA (8)
**2.7 Vitamin**	☐ Yes (1) ☐ No (0) – If no, skip to 2.8 ☐ NR (9)
**2.7.a Multivitamin with iron**	☐ Yes (1) ☐ No (0) ☐ NR (9)
**2.7.b Multivitamin without iron**	☐ Yes (1) ☐ No (0) ☐ NR (9)
**2.7.c Iron**	☐ Yes (1) ☐ No (0) ☐ NR (9)
**2.7.d Calcium**	☐ Yes (1) ☐ No (0) ☐ NR (9)
**2.7.e Vitamin D**	☐ Yes (1) ☐ No (0) ☐ NR (9)
**2.7.f Other**	☐ Yes (1) ☐ No (0) ☐ NR (9)
**2.8 Current Growth Parameters**	
**2.8.a.i Weight**	☐☐☐.☐ kilograms ☐ NR (9)
**2.8.a.ii Length or Height**	☐☐☐.☐ cm ☐ NR (9)
**2.8.b.i Length or Height z-score**	Value: ___ ☐.☐ ☐ NR (9)
**2.8.b.ii BMI z-score**	Value: ___ ☐.☐ ☐ NR (9) ☐ NA (8)
**2.8.b.iii Weight for length z-score**	Value: ___ ☐.☐ ☐ NR (9) ☐ NA (8)
**2.9 Evidence of malnutrition**	
**2.9.a Evidence of malnutrition- height/length z-score**	☐ None (0) ☐ Severe (1) ☐ NR (9)
**2.9.b Evidence of malnutrition BMI z-score**	☐ None (0) ☐ Mild (1) ☐ Moderate (2) ☐ Severe (3) ☐ NR (9) ☐ NA (8)
**2.9.c Evidence of malnutrition weight for length z-score**	☐ None (0) ☐ Mild (1) ☐ Moderate (2) ☐ Severe (3) ☐ NR (9) ☐ NA (8)

**2.9.d Evidence of malnutrition weight gain velocity decline (< 2 years of age)**	☐ None (0) ☐ Mild (1) ☐ Moderate (2) ☐ Severe (3) ☐ NR (9) ☐ NA (8)
**2.9.e Evidence of malnutrition experiencing weight loss >5% over 3 months (2 to 20 years)**	☐ None (0) ☐ Mild (1) ☐ Moderate (2) ☐ Severe (3) ☐ NR (9) ☐ NA (8)
**2.9.f Evidence of malnutrition deceleration in BMI or weight for length**	☐ None (0) ☐ Mild (1) ☐ Moderate (2) ☐ Severe (3) ☐ NR (9) ☐ NA (8)
**2.9.g Evidence of malnutrition due to inadequate caloric intake**	☐ None (0) ☐ Mild (1) ☐ Moderate (2) ☐ Severe (3) ☐ NR (9) ☐ NA (8)

SECTION 2: NUTRITION DOMAIN PROTOCOL	
Item	Detail
2.1	These are the raw data elements that will provide information to determine the following additional data points: primary source of nutrition, current and percent supplementation, and evidence of dependence on supplementation.
2.1.a	Total calories from tube and oral, fluid and food.
2.1.b	Total calories from tube feeding
2.1.c	Total calories from formula consumed orally
2.1.d	Total calories from food consumed orally
2.1.e	Total calories from fluid that is not formula
2.1.f	Estimated daily calorie needs – if range is provided by RD, then use the midpoint
2.2	Classifies the primary source of nutrition for the patient. Primary refers to consuming > 50% of total energy and nutrition needs from a specific mode of intake. This is calculated from Total Calories (2.1.a) and not from Estimated Needs (2.1.f). Determination of source of nutrition should be based on nutrition evaluation by an RDN gathered via 24-hour Dietary Recall method and supported by clinical interview. Conduct this as a true 24-hour recall if possible. If yesterday was an atypical day, have parents provide information from the most recent typical day.<br><br>i. Tube feeding – Refers to a medical device used to provide nutrition placed either through the nose (e.g., nasogastric, nasoduodenal, nasojejunal) or directly in the abdomen (e.g., gastrostomy, gastrojejunostomy, jejunostomy).<br><br>ii. Drinking formula – Refers to formula supplementation with a nutritionally complete drink (i.e., contains essential vitamins, minerals, protein, carbs, etc.) delivered through any format (e.g., bottle, sippy cup, straw). This excludes cases involving fluid that does not provide complete nutrition (e.g., cow milk or calorie fortified milk).<br><br>iii. Oral consumption – Refers to nutrition that occurs by eating food regardless of the texture or variety of the food items. This includes fluids other than formula
2.3	Nutritional Supplementation
2.3.a	Identifies whether the patient requires supplementation involving a nutritionally complete fluid by tube (no age limitations), by IV (no age limitations) or oral (over the age of 1 year adjusted). Supplementation does not require formula to represent the primary source of nutrition and there may be cases where supplementation is used to address underlying nutritional deficits or gaps (e.g., one bottle of a nutritionally complete fluid per day), but not associated with volume needs. This does not include high calorie milk – the supplement must be nutritionally complete.
2.3.b	Estimates the percentage of a patient's average daily caloric needs that are obtained via supplementation as determined by nutritional analysis completed by the RDN. This is calculated from Total Calories (2.1.a) and not from Estimated Needs (2.1.f).
2.3.c	Based on today's response, the reason for supplementation is:<br>To promote growth- Refers to whether the supplementation is related to volume to maintain/sustain growth (i.e., > 50% by tube or oral intake) or prevent a diagnosis of malnutrition (i.e. weight-for-age or BMI z-score < -1).<br>To address nutritional needs- Refers to whether the supplementation is needed to meet macro and/or micronutrient needs.<br>To support hydration- Refers to whether the supplementation is needed to maintain hydration
2.3.d	Based on today's response, identifies the source of the supplementation – i.e., formula via tube or oral; IV fluids.
2.4	Dietary diversity should be determined by an RDN based on the 24-hour Dietary Recall method and the clinical interview, focusing only on oral intake (if child is exclusively tube fed, responses would be 0 for each item). During the clinical interview, the RDN provides caregivers with the opportunity to identify any additional food not included in the 24-hour time period. Specific prompts include (1) reporting items that

	the child "consistently and currently accepts" (i.e., within the last month) with "minimal prompting or coaxing" in each food group and (2) describe food items consumed at meals "during a typical weekday and weekend."<br><br>Specifications:<br>- Mixed dish items (e.g., macaroni and cheese) should be categorized based on the dominant food group represented in the combined item and only counted once.<br>- Single foods (e.g., avocado, tomato, corn) that could be categorized as two different food groups should only be counted in one group at the RDNs discretion.<br>- Juice does not count as fruit<br>- Milk counts as dairy/dairy alternative<br>- Formula counts as dairy/dairy alternative<br><br>2.4.a – 2.4.f: Based on these data, the RDN 1) records whether the patient consumes a food item from each of the five food groups (i.e., vegetable, fruits, dairy, proteins, grains) and 2) estimates the total number of items within each group using a scale (i.e., 1-2 items, 3-4 items, 5 or more).
2.5	Evidence of nutritional deficiency as supported by laboratory analysis (blood work identifying nutritional deficiencies)<br>Note: If lab work ordered at or after the time of the assessment, these data should not be considered. Only use if within 6 months prior to visit. Use N/A if lab analysis was not conducted at all prior to the visit
2.6	Analysis of the 24-recall should be completed using an established food and ingredient database (e.g., ESHA's Food Processor Nutrition Analysis software). Data collection focused on intake of 13 nutritional areas: energy, carbohydrates, and protein; vitamins A, B-12, C, D, and E; folic acid; calcium; iron; zinc; protein. This selection of nutrients is consistent with previous research focusing on dietary status in children with pediatric feeding disorders. Nutrient inadequacy is defined relative to dietary reference intake (DRI). To identify children whose intake of documented nutrients is less than required to support health, we recommend risk of nutrient inadequacy be set at < 80% of DRI. The CRF identifies a DRI <80% by nutrient. This is based on food and fluid sources alone (i.e., not including multivitamin)<br><br>In clinical practice this would not be run if on supplemental tube feeding, supplemental oral formula, or multivitamin. As such, should only consider food and beverages without formula and tube feeding.
2.7	Vitamins. Endorse Yes or No for Multivitamin, Iron, Calcium, and Vitamin D
2.8	Growth parameters collected on the date of the appointment include height or length and weight. Body mass index (BMI) or weight for length is calculated. Measurements should be referenced against sex- and age-specific clinical growth charts (CDC or WHO) and translated into z-scores.<br><br>Data entry for each anthropometric parameter below (2.8.a.i through 2.8.c.iii) involves specifying the relative value of the z-score (i.e., positive or negative).
2.8.a.i	Weight: Patient weight should be recorded using a digital scale that measures to a precision of 0.1 kg.
2.8.a.ii	Length or Height: Patients less than 2 years of age should be measured lying down (recumbent length). Measurement of height for patients 2 or older should involve a wall-mounted stadiometer. Both measurements should be to a precision of .1 meters.
2.8.b.i	Length or Height z-score.
2.8.b.ii	BMI z-score
2.8.b.iii	Weight for length z-score
2.9	Evidence for and classification of malnutrition based on height z-score, BMI z-score, weight for length z-score, growth curve analysis, and caloric intake. Each item is scored individually and there will not be an overarching yes/no for malnutrition. When entering data, need to be mindful of using N/A based on the child's age.
2.9.a	Evidence of malnutrition (height). Stunting of height is a well-established sign of chronic malnutrition, which is defined by the WHO as a height-for-age z-score of -3 or greater (severe).
2.9.b	Evidence of malnutrition BMI z-score. Classification of malnutrition based on BMI-for-age is defined as:

	Mild malnutrition: -1.0 to -1.9 z score<br>Moderate malnutrition: -2.0 to -2.9 z score<br>Severe malnutrition: - 3.0 or greater z score
2.9.c	Evidence of malnutrition weight for length z-score. Classification of malnutrition based on weight for length z-score is defined as:<br>Mild malnutrition: -1.0 to -1.9 z score<br>Moderate malnutrition: -2.0 to -2.9 z score<br>Severe malnutrition: - 3.0 or greater z score
2.9.d	Growth trajectory below two years of age focuses on expected weight gain velocity based on a percentage of the norm, with 75% representing the threshold to trigger concerns with weight gain velocity.<br><br>Classification of weight gain velocity severity based on consensus recommendation for classification of undernutrition severity defined as:<br>Mild malnutrition: Less than 75% of the norm for expected weight gain<br>Moderate malnutrition: Less than 50% of the norm for expected weight gain<br>Severe malnutrition: Less than 25% of the norm for expected weight gain
2.9.e	Growth trajectory above two years includes weight loss in a child (2-20 years of age) flagged when the threshold exceeds 5% of usual body weight.<br><br>Classification of weight loss severity based on consensus recommendation for classification of undernutrition severity defined as:<br>Mild malnutrition: 5% usual body weight<br>Moderate malnutrition: 7.5% usual body weight<br>Severe malnutrition: 10% usual body weight
2.9.f	Deceleration in weight for length or BMI-for-Age z score provides a sign of chronic malnutrition, with the threshold set at 1 z score decline to trigger clinical attention.<br><br>Classification of deceleration in weight/height severity based on consensus recommendation for classification of undernutrition severity defined as:<br>Mild malnutrition: -1.0 to -1.9 z score<br>Moderate malnutrition: -2.0 to -2.9 z score<br>Severe malnutrition: - 3.0 or greater z score
2.9.g	Malnutrition due to inadequate caloric intake<br><br>Classification is defined as:<br>Mild malnutrition: 51-75% estimated energy/protein needs<br>Moderate malnutrition: 26-50% estimated energy/protein needs<br>Severe malnutrition: ≤25% estimated energy/protein needs
