# Supplementary material for: An updated framework for characterizing patients with pediatric feeding disorder
Source: Front Child Adolesc Psychiatry. 2025 Sep 15;4:1653288. doi: 10.3389/frcha.2025.1653288 (PMC12477044; doi:10.3389/frcha.2025.1653288)
Supplement: Supplementary file 4 [file Image4.pdf]

Supplementary Figure 4. Feeding Skill Case Report Form

SECTION 3: FEEDING SKILL DOMAIN		
**3.1.a Past feeding therapy**	☐ Yes (1) ☐ No (0) – If no, skip to 3.2 ☐ NR (9)	
**3.1.b Provider type**	Speech Language Pathologist	☐ Yes (1) ☐ No (0) ☐ NR (9)
	Occupational Therapist	☐ Yes (1) ☐ No (0) ☐ NR (9)
	Psychologist	☐ Yes (1) ☐ No (0) ☐ NR (9)
	Board Certified Behavior Analyst	☐ Yes (1) ☐ No (0) ☐ NR (9)
	Other	☐ Yes (1) ☐ No (0) ☐ NR (9)
	Other specify:	
**3.1.c Estimated duration (months)**	☐ <1 – 3 months (4) ☐ 4– 6 months (3) ☐ 7 – 11 months (2)	
	☐ 12 months+ (1) ☐ NR (9) ☐ NA (8)	
**3.2.a Estimated average meal duration solids and liquids**	☐☐ minutes | ☐ NR (9) | ☐ NA (8)	
**3.2.b Estimated average meal duration liquids only**	☐☐ minutes | ☐ NR (9) | ☐ NA (8)	
**3.3.a Is the child demonstrating chronologically age expected oral skill?**	☐ Yes (1) ☐ No (0) ☐ NR (9)	
**3.3.b Is the child demonstrating overt or soft signs of aspirations?**	☐ Yes (1) ☐ No (0) ☐ NR (9)	
**3.4 Type of texture currently consumed**	i. Pureed food (IDDSI level 4)	☐ Yes (1) ☐ No (0) ☐ NA (8) ☐ NR (9)
	ii. Fork mashed food (IDDSI level 5)	☐ Yes (1) ☐ No (0) ☐ NA (8) ☐ NR (9)
	iii. Soft solid (IDDSI level 6)	☐ Yes (1) ☐ No (0) ☐ NA (8) ☐ NR (9)
	iv. Chewable food (IDDSI level 7)	☐ Yes (1) ☐ No (0) ☐ NA (8) ☐ NR (9)
**3.5.a Modified feeding support**	☐ Yes (1) ☐ No (0) – If no, skip to 3.6 ☐ NA (8) ☐ NR (9)	
**3.5.b Type of feeding support**	i. Special therapeutic seating	☐ Yes (1) ☐ No (0) ☐ NA (8) ☐ NR (9)
	ii. Altered feeding position	☐ Yes (1) ☐ No (0) ☐ NA (8) ☐ NR (9)
	iii. Added support for stability	☐ Yes (1) ☐ No (0) ☐ NA (8) ☐ NR (9)
**3.6.a Modified feeding strategies**	☐ Yes (1) ☐ No (0) – If no, skip to 3.7 ☐ NA (8) ☐ NR (9)	
**3.6.b Type of feeding strategies**	i. Altered bite placement	☐ Yes (1) ☐ No (0) ☐ NA (8) ☐ NR (9)
	ii. Use of special utensil	☐ Yes (1) ☐ No (0) ☐ NA (8) ☐ NR (9)
	iii. Use of special bottle	☐ Yes (1) ☐ No (0) ☐ NA (8) ☐ NR (9)
	iv. Other presentation method	☐ Yes (1) ☐ No (0) ☐ NA (8) ☐ NR (9)
	Other specify:	
**3.7.a Self-feeding**	☐ Yes (1) ☐ No (0) – If no, skip to 3.8 ☐ NA (8) ☐ NR (9)	
**3.7.b Type of self-feeding**	i. Spoon use	☐ Yes (1) ☐ No (0) ☐ NA (8) ☐ NR (9)
	ii. Fork use	☐ Yes (1) ☐ No (0) ☐ NA (8) ☐ NR (9)
	iii. Finger feeding	☐ Yes (1) ☐ No (0) ☐ NA (8) ☐ NR (9)
**3.8.a Drinking liquids**	☐ Yes (1) ☐ No (0) ☐ Not permitted (2) ☐ NR (9)	
**3.8.b Type of drinking format**	i. Breastfeeding/chestfeeding	☐ Yes (1) ☐ No (0) ☐ NR (9)
	ii. Bottle	☐ Yes (1) ☐ No (0) ☐ NR (9)

	iii. Sippy cup	☐ Yes (1) ☐ No (0) ☐ NR (9)
	iv. Open cup	☐ Yes (1) ☐ No (0) ☐ NR (9)
	v. Straw	☐ Yes (1) ☐ No (0) ☐ NR (9)
	vi. Sports cap bottle	☐ Yes (1) ☐ No (0) ☐ NR (9)
	vii. Other	☐ Yes (1) ☐ No (0) ☐ NR (9)
	Other specify:	
**3.8.c Is liquid thickened for safety?**	☐ Yes (1) ☐ No (0) ☐ NR (9)	
**3.8.d Drinking independently?**	☐ Yes (1) ☐ No (0) ☐ NR (9)	

SECTION 3: FEEDING SKILL DOMAIN PROTOCOL	
Item	Detail
3.1.a	Refers to participation in any therapeutic activities lasting 1 session or more targeting the patient's PFD, with a primary focus of modifying feeding skill and/or improving intake, variety, and/or tolerance of food or liquid. This excludes assessment or initial intake.
3.1.b	Identifies the discipline of the provider who delivered feeding therapy. If the patient has experience with multiple provider types, select all disciplines involved in providing feeding treatment.
3.1.c	Estimates the approximate length of time the patient participated in feeding therapy and places this time within five periods anchored by 1-3 months to 12 months or greater. If the patient participated in multiple feeding therapies, the total duration should be tallied and placed in the appropriate timeframe.
3.2.a	The average duration of meals including solids and liquids (in minutes) is an estimate provided by the caregiver. If a range is provided (e.g., anywhere from 10 to 45 minutes), the caregiver is asked to provide the most common duration of mealtimes. If the caregiver continues to provide a range, the provider records upper limit of the range.<br><br>If intake only occurs outside of structured meals (i.e., grazing), enter N/A for this item.
3.2.b	The average duration of only oral caloric liquid meals (in minutes) is an estimate provided by the caregiver. If a range is provided (e.g., anywhere from 10 to 45 minutes), the caregiver is asked to provide the most common duration of mealtimes. If the caregiver continues to provide a range, the provider records upper limit of the range.<br><br>If liquids are included in meals or the child only drinks water for hydration, enter N/A for this item. If intake only occurs outside of structured meals (i.e., grazing), enter N/A for this item.
3.3.a	This item asks the SLP to make a determination at time of visit as to whether a child is demonstrating chronologically age expected oral skill (this item does not consider volume or variety of food consumed). If not reported or observed can reply "no" because child is not demonstrating.
3.3.b	This item asks the SLP to make a determination at time of visit as to whether a child is presenting with concerns for swallowing safety (this item does not consider volume or variety of food consumed).
3.4	Refers to the texture of food a child currently eats. Endorse highest level; it is acceptable if child only eats 1 food at that level.<br>i. Pureed food (IDDSI level 4) – Diet includes food(s) of a pureed/smooth texture<br>ii. Fork mashed food (IDDSI level 5) – Diet involves food(s) mechanically altered with fork (or similar utensil)<br>iii. Soft solid (IDDSI level 6) – Diet involves soft and moist foods that can be easily mechanically altered with fork (or similar utensil)<br>iv. Chewable food (IDDSI level 7) – Diet includes food(s) that require mastication<br><br>Endorse N/A if no oral feeding
3.5.a	Identifies whether the child requires some form of modification in the feeding environment in line with PFD diagnostic criteria 3.b (i.e., use of modified feeding position or equipment).<br><br>Note: This excludes use of seating to manage behavioral concerns, such as use of a booster seat with a buckle to promote remaining seated at the table.
3.5.b	Details the type of environmental modification currently employed during meals, which include:

	i. Special therapeutic seating – Use of adaptive feeding chair to improve postural control and stability due to a child's abnormal muscle control.<br>ii. Altered feeding position – Modifying the angle of the child during meals by reclining the chair back or holding the child in a reclined position in the feeder's lap.<br>iii. Added support for stability – Use of towels, cushions, or other types of wedge with a seat to improve postural control and stability due to a child's abnormal muscle control.
3.6.a	Identifies whether the child requires some form of modification in the delivery of food and/or liquid in line with PFD diagnostic criteria regardless of intent 3.c – i.e., use of modified feeding strategies including slow flow nipple for preemie infant.
3.6.b	Details the type of modified feeding strategy currently employed during meals, which include:<br>i. Altered bite placement – Placement of bolus at a specific location in the mouth (e.g., cheeks, directly on tongue) or different placement of bolus on the spoon (e.g., front loading the bolus) to assist with consumption of food/liquid<br>ii. Use of special utensil – Current use of a therapeutic utensil to present food/liquid during meals<br>iii. Use of special bottle – Current use of adaptive bottle that controls flow and deposit of liquid during feeding<br>iv. Other presentation method – Refers to other adaptive tools/techniques employed due to concerns about swallow safety or poor oral skill
3.7.a	Self-feeding involves a child's <u>ability</u> to independently deposit bites of food using utensils (e.g., spoon, fork) or fingers.
3.7.b	Details the method(s) of self-feeding, which include:<br>i. Spoon use – Child demonstrates the ability to use a spoon to feed him/herself<br>ii. Fork use – Child demonstrates the ability to use a fork to feed him/herself<br>iii. Finger feeding – Child demonstrates the ability to use fingers to feed him/herself<br><br>*Child does not have to self-feed in meals consistently to be marked as Yes (1) for this question, but simply must demonstrate capability to do so.*
3.8.a	Drinking liquids may involve drink delivery in a non-self-feeder (caregiver presented bites) or self-feeder (child independent consumption) format; consider ability to drink regardless of quantity consumed. A positive ("Yes") on this item reflects that the patient engages in drinking with a specific modality (e.g., bottle, sippy cup).
3.8.b	Details the drinking format that fluid is delivered (self or by caregiver), which include:<br>i. Breastfeeding/chestfeeding – Receiving human milk from lactating parent that requires sucking motion to access liquid<br>ii. Bottle – Baby bottle or equivalent with a nipple that requires a sucking motion to access liquid<br>iii. Sippy cup – Cup with a lid and spout intended for use with an infant or young child (includes 360 cups)<br>iv. Open cup – Cup without a lid or straw (includes nosey/flexi-cut cup, standard soda or water bottle without sports bottle top)<br>v. Straw – Use of straw utensil to carry contents of liquid to the mouth (includes honey bear with negative oral pressure to extract)<br>vi. Sports cap bottle – Bottle with spout designed to aid drinking without spilling, the liquid can be squirted or squeezed into the mouth or may include bite valve such as a CamelBak<br>vii. Other – Provides opportunity to document other liquid delivery methods such as those intended for modified flow rate from dysphagia perspective (includes reflo smart and honey bear not requiring negative oral pressure to extract)
3.8.c	Specifies if the liquid is thickened to any degree. Only endorse if liquid is thickened for oral control and/or safety (versus other reasons, such as an attempt to control reflux).

3.8.d	Identifies that the child has the ability to independently deposit and consume the drink regardless of type of drinking format

**Note: For all items, consider where the child is at the time of the visit (i.e., how they walk in) and not what would be recommended
