# Supplementary material for: An updated framework for characterizing patients with pediatric feeding disorder
Source: Front Child Adolesc Psychiatry. 2025 Sep 15;4:1653288. doi: 10.3389/frcha.2025.1653288 (PMC12477044; doi:10.3389/frcha.2025.1653288)
Supplement: Supplementary file 5 [file Image5.pdf]

Supplementary Figure 5. Psychosocial Case Report Form

SECTION 4: PSYCHOSOCIAL DOMAIN		
**4.1 Child avoidance behaviors**	☐ Yes (1) ☐ No (0) – If no, skip to 4.2 ☐ NR (9)	
**If yes, check all that apply:**	Turns head away from food	☐ Yes (1) ☐ No (0) ☐ NR (9)
	Pushes away food	☐ Yes (1) ☐ No (0) ☐ NR (9)
	Throws food/plate/utensil	☐ Yes (1) ☐ No (0) ☐ NR (9)
	Cries/screams/tantrums during meals	☐ Yes (1) ☐ No (0) ☐ NR (9)
	Spits food out of mouth	☐ Yes (1) ☐ No (0) ☐ NR (9)
	Aggression toward feeder	☐ Yes (1) ☐ No (0) ☐ NR (9)
	Leaves the table	☐ Yes (1) ☐ No (0) ☐ NR (9)
	Lack of interest in eating/dawdles	☐ Yes (1) ☐ No (0) ☐ NR (9)
	Coughs	☐ Yes (1) ☐ No (0) ☐ NR (9)
	Gags	☐ Yes (1) ☐ No (0) ☐ NR (9)
	Vomits	☐ Yes (1) ☐ No (0) ☐ NR (9)
	Lip pursing	☐ Yes (1) ☐ No (0) ☐ NR (9)
	Teeth clenching	☐ Yes (1) ☐ No (0) ☐ NR (9)
	Packing/pocketing food	☐ Yes (1) ☐ No (0) ☐ NR (9)
**4.2 Caregiver management approach**	☐ Yes (1) ☐ No (0) – If no, skip to 4.3 ☐ NR (9)	
**If yes, check all that apply:**	Excessive caregiver attention	☐ Yes (1) ☐ No (0) ☐ NR (9)
	Excessive caregiver support	☐ Yes (1) ☐ No (0) ☐ NR (9)
	Reinforcement procedures	☐ Yes (1) ☐ No (0) ☐ NR (9)
	Punishment procedures	☐ Yes (1) ☐ No (0) ☐ NR (9)
	Forced feeding	☐ Yes (1) ☐ No (0) ☐ NR (9)
	Only offering preferred foods	☐ Yes (1) ☐ No (0) ☐ NR (9)
	Replacing refused food with preferred food	☐ Yes (1) ☐ No (0) ☐ NR (9)
	Providing access to food/drink outside meal/snack schedule	☐ Yes (1) ☐ No (0) ☐ NR (9)
	Distracting feeding environment	☐ Yes (1) ☐ No (0) ☐ NR (9)
	Ending the meal	☐ Yes (1) ☐ No (0) ☐ NR (9)
	No longer conduct meals	☐ Yes (1) ☐ No (0) ☐ NR (9)
	Peer or caregiver modeling	☐ Yes (1) ☐ No (0) ☐ NR (9)
	Scheduling for appetite	☐ Yes (1) ☐ No (0) ☐ NR (9)
**4.3 Disruptions in social functioning**	☐ Yes (1) ☐ No (0) – If no, skip to 4.4 ☐ NR (9)	
**If yes, check all that apply:**	Family meals	☐ Yes (1) ☐ No (0) ☐ NR (9)
	Social events	☐ Yes (1) ☐ No (0) ☐ NR (9)
	Eating location	☐ Yes (1) ☐ No (0) ☐ NR (9)
	School	☐ Yes (1) ☐ No (0) ☐ NR (9) ☐ NA (8)
**4.4 Disruption in parent-child relationship**	☐ Yes (1) ☐ No (0) – If no, skip to 4.5 ☐ NR (9)	
**If yes, check all that apply:**	Poor mealtime interactions	☐ Yes (1) ☐ No (0) ☐ NR (9)
	Disengagement from the meal	☐ Yes (1) ☐ No (0) ☐ NR (9)
	Caregiver stress	☐ Yes (1) ☐ No (0) ☐ NR (9)
**4.5 Behavioral/Developmental Complexity**	History of behavioral services	☐ Yes (1) ☐ No (0) ☐ NR (9)
	History of developmental services	☐ Yes (1) ☐ No (0) ☐ NR (9)
	Problem behaviors outside of meals (externalizing)	☐ Yes (1) ☐ No (0) ☐ NR (9)
	Problem behaviors outside of meals (internalizing)	☐ Yes (1) ☐ No (0) ☐ NR (9)

SECTION 4: PSYCHOSOCIAL DOMAIN PROTOCOL	
Item	Detail
4.1	Child avoidance behaviors are those that interfere with consumption of food. Endorse if child demonstrates the behavior at time of intake.
4.2	The strategies listed below represent commonly observed management strategies used by caregivers at mealtimes. Endorse if caregiver demonstrates this behavior at the time of intake.<br><br>Excessive caregiver attention – Reflects the use of repeated prompting, coaxing and/or comforting during meals aimed at getting the child to eat and/or try new foods, which may unintentionally reinforce food refusal.<br>Excessive caregiver support – Denotes that consumption is reliant on caregiver involvement beyond a child's required developmental accommodations associated with developmental or neuromuscular delays, including cases in which a caregiver feeds most/all bites despite the child having the skill to self-feed or the required presence of the caregiver during meals for the child to consistently consume food.<br>Reinforcement procedure- Includes providing praise, preferred items, or activities/privileges following the child's appropriate mealtime behavior (e.g., taking a bite, swallowing a bite).<br>Punishment procedure – Includes the use of verbal reprimands, negative punishment (e.g., placing in time-out; restricting access to preferred activities during or outside of meals) and positive punishment (e.g., spanking) in response to a child's problematic mealtime behaviors or lack of eating.<br>Forced feeding – Refers to a level of persisting with bite presentations that involves some form of physical restraint (e.g., holding arms) and placement of food into the mouth despite the child's protest.<br>Only offering preferred foods – Refers to ceasing attempts to introduce new or non-preferred food items, which limits a child's exposure and opportunity to expand his/her diet.<br>Replacing refused food with preferred foods – Involves the caregiver removing new, novel, or non-preferred food and replacing it with preferred food items when faced with rejection/refusal to eat.<br>Providing access to food/drink outside of the meal/snack schedule – Reflects grazing, or allowing the child free access to fluid throughout the day, which can cause a sense of satiety<br>Distracting feeding environment – Providing access to tangible item (e.g., watching a video, toy play) non-contingently as a means to distract or pacify potential distress and/or refusal.<br>Ending the meal in response to child protest – Involves the caregiver removing the food and associated feeding demand, including allowing the child to leave the table.<br>No longer conducting meals- Refers to cases in which the caregiver reports no longer conducting meals or attempting to expand the volume or variety of food consumed during meals.<br>Peer or caregiver modeling- Caregiver intentionally places child in a situation to encourage peer modeling of good eating or caregiver intentionally shows their child appropriate eating behavior by engaging in this behavior themselves and pointing this out to the child.<br>Scheduling for appetite- Intentionally manipulate feeding schedule (tube/oral) to promote hunger.
4.3	Indicates that the feeding dysfunction affects a child's participation or restriction in daily activities associated with eating. Endorse if this occurs at time of intake.<br><br>Family meals – Refers to cases where a family has ceased or significantly reduced eating together as a unit due to chronic and persistent food refusal.<br><br>Social events – Refers to limitations in a child's involvement in events outside the home (e.g., birthday parties) that involve food and/or require eating.<br><br>Eating locations – Refer to restriction in the location(s) that a child will consistently consume food, such as only eating at home and rejecting food at restaurants or other outside settings.<br><br>School – Refers to limitations in a child's participation in activities associated with eating (e.g., meals, snack) in the school setting.
4.4	Indicates that the feeding dysfunction affects the caregiver-child relationship during or outside of meals. Endorse if this occurs at time of intake.

	<u>Poor mealtime interactions</u> – Refers to frequent arguments, mealtime disruptions, high negative statements by child and/or caregivers, and/or frustration and/or anger associated with food rejection.<br><br><u>Disengagement from the meal</u> – Refers to withdrawal of caregiver or child from the meal as reflected by caregiver or child leaving the meal environment, not sitting together while eating, and/or not interacting during the meal.<br><br><u>Caregiver stress</u> – Refers to acknowledged worry, anxiety, dread, and/or despair by the caregiver associated with presenting food and/or conducting meals in both the home and/or public settings.
4.5	This item serves as a proxy for a child's behavioral and/or developmental complexity as reflected by a history of receiving therapeutic and/or educational support for issues not directly attributable to a child's mealtime performance, as well as assesses caregiver concern about the presence of problem behavior outside of meals.<br><br><u>i. History of behavioral services</u> – Involves current or past therapy to address behavioral issues outside of meals such as non-compliance, disruptive behavior, aggression, self-injury, inattention.<br><br><u>ii. History of developmental services</u> – Involves current or past educational/developmental support (e.g., early intervention, special education) associated with concerns regarding a delay.<br><br><u>iii. Problem behaviors outside of meals</u> (externalizing) – Refers to problem behavior (e.g., non-compliance, disruptive behavior, aggression, self-injury, inattention) that caregivers report interfere with a child's functioning in the family, school, or community.<br><br><u>iv. Problem behaviors outside of meals</u> (internalizing) – Refers to problem behavior (e.g., anxiety, depression) that caregivers report interfere with a child's functioning in the family, school, or community.
